# Increasing ecological validity in mental fatigue research—A Footbonaut study

**DOI:** 10.3389/fpsyg.2025.1586944

**Published:** 2025-05-27

**Authors:** Helena Weiler, Fabienne Ennigkeit, Jan Spielmann, Chris Englert

**Affiliations:** ^1^Department of Sport Psychology, Institute of Sports Sciences, Goethe-University Frankfurt, Frankfurt, Germany; ^2^TSG Research Lab gGmbH, Zuzenhausen, Germany

**Keywords:** mental fatigue, mental effort, technical performance, cognitive performance, soccer, team sport

## Abstract

**Introduction:**

Past studies have mainly used Stroop tasks to induce mental fatigue in soccer. However, due to the non-sport-specificity of these tasks, their transferability to the real-life effects of mental fatigue in soccer have been questioned. The study's aim was to investigate the effects of two different versions (mentally less vs. mentally more demanding) of a soccer passing task in the so-called Footbonaut on cognitive and soccer-specific performance.

**Methods:**

A randomized, counterbalanced experimental within-subjects design was employed (*N* = 27). We developed two different versions of the soccer passing task in the Footbonaut: a mentally more demanding decision-making and inhibition task in the experimental condition, and a mentally less demanding standard task of the Footbonaut in the control condition.

**Results:**

Participants showed significantly worse soccer-specific performance in the experimental condition compared to the control condition. No corresponding effects were revealed in cognitive performance.

**Discussion:**

The findings suggest that cognitive-motor interference induced by 30-min Footbonaut technology-based training may induce mental fatigue in soccer players. Future studies should consider developing mentally less-demanding yet comparable control tasks.

## 1 Introduction

“*The only explanation now is that we are a fatigued team, mentally more or less.” – Klopp blames mental fatigue for Liverpool loss to Brighton* (Poole, 2021).

Soccer, apart from its physical demands, is also an extremely cognitively and mentally demanding sport (Thompson et al., 2019). While playing, soccer players must remain focused over long periods of time, only paying attention to the situationally relevant while suppressing distracting information (e.g., fan chants from the crowd; Kunrath et al., 2020a). Additionally, accurate decisions must be made under strict time and space constraints, while also sticking to certain team tactics (Angius et al., 2022; Gantois et al., 2020; García-Calvo et al., 2021; Thompson et al., 2019). Furthermore, players must deal with several other aspects that operate outside the game and are psychologically challenging (e.g., tightly-timed playing schedules, media interest, frequent traveling; Smith et al., 2018; Thompson C. J. et al., 2020). Dealing with these soccer-specific and unspecific mental demands over extended periods of time requires high levels of mental effort and can subsequently cause mental fatigue in soccer players (e.g., Díaz-García et al., 2023; García-Calvo et al., 2021).

According to the psychobiological model (Marcora et al., 2009), mental fatigue is defined as a psychobiological state that is induced by prolonged periods of mentally demanding activity that leads to changes in several subjective (e.g., reduced motivation, increased perception of effort; Van Cutsem and Marcora, 2021), physiological (e.g., changes in heart rate; Englert and Wolff, 2015) and behavioral parameters (e.g., slower reaction times; Englert and Bertrams, 2014a). Especially, the detrimental effects of mental fatigue on physical (e.g., reduced running distance within a YoYo Intermittent Recovery test; Smith et al., 2016), technical (e.g., performing less accurate and slower passes in the Loughborough Soccer Passing Test (LSPT); Grgic et al., 2022), tactical (e.g., reduction in team synchronization during a Small-Sided-Game (SSG); Coutinho et al., 2017) and cognitive performance (e.g., impairment of passing decision-making performance; Gantois et al., 2020) have been consistently empirically demonstrated in previous research.

The dominant research approach for investigating these effects of mental fatigue is the sequential dual-task paradigm (Englert and Bertrams, 2014b; see also Sun et al., 2022). The independent variable, i.e., mental fatigue, is experimentally manipulated in the first task: In the experimental condition, participants perform a mentally demanding task (e.g., Stroop task), while in the control condition, participants are confronted with a mentally less demanding task (e.g., reading magazines). The subsequent secondary task (the dependent variable; e.g., handgrip task, cycling test) is identical for both conditions and serves to investigate potential performance-related differences between the two conditions (Marcora et al., 2009). It has been shown that mentally fatigued individuals tend to perform worse in the secondary task (for two recent meta-analyses, see also Brown et al., 2019; Giboin and Wolff, 2019). While several studies have adopted sport-specific tasks as the secondary task (e.g., Giboin and Wolff, 2019; Pageaux and Lepers, 2018), sport-specific tasks as manipulation tasks have not yet been addressed to the same extent. This leads to a lack of ecological validity in the corresponding research field (Bian et al., 2022; Coutinho et al., 2017; Musculus et al., 2022; Thompson et al., 2019). In particular, this applies to the (modified) Stroop task, which has been used repeatedly as a manipulation task to investigate the role of mental fatigue in sport-specific contexts (Thompson et al., 2019). The Stroop task requires participants to suppress a dominant response tendency (i.e., to ignore the semantic meaning of a certain color word) and to show a different response instead (i.e., to name the font color; e.g., when the word “red” is written in yellow font color, the correct answer would be “yellow”). While inhibitory control is also relevant in several types of sport, the Stroop task itself is by no means sport-specific, raising the question of to what extent lab-based findings on mental fatigue are transferable to the applied field (Kunrath et al., 2020b).

Several researchers have called for developing and validating ecologically valid (sport-specific) mentally fatiguing tasks to gain a better understanding of the importance and relevance of mental fatigue in sports (e.g., Bian et al., 2022). According to Musculus et al. (2022) and Thompson et al. (2019), the level of ecological validity can be increased by (1) increasing the sport specificity of the respective task (i.e., soccer specificity) and (2) by contextual interference of the tasks implemented. In line with Musculus et al. (2022), the level of sport-specificity of a given task is determined by its setting (comparable space of movement, e.g., 360°), the stimulus presented (presenting stimuli relevant to the sport, e.g., pictures or video simulations, receiving a ball) and the required response (e.g., moving in a similar manner as during an actual game). Contextual interference means increased task engagement, which is achieved for example through constant problem solving based on ever-changing task constraints, instead of being confronted with repeatedly performing the same task (e.g., Stroop task; Thompson et al., 2019). Further, an increase in sport specificity and contextual interference enables a better differentiation between mental fatigue and related constructs, such as boredom (Wolff et al., 2021). Previous research has shown that performing a monotonous task over an extended period can lead to increased sensations of boredom (Radtke et al., 2023; Thompson C. et al., 2020). Forcing oneself to keep working on a boring task requires mental effort and might trigger mental fatigue. Therefore, tasks designed to induce mental fatigue should be interesting enough to not induce sensations of increased boredom, which appears to be more likely in sport-specific tasks (Martarelli et al., 2023).

Several researchers have tried to gain a better understanding of the factors potentially leading to increased sensations of mental fatigue in sports (e.g., Díaz-García et al., 2021). For instance, in several studies athletes were interviewed and asked which non-specific (e.g., media engagement; Russell et al., 2019; Weiler et al., 2025) and sport-specific demands (Bian et al., 2025; Thompson C. J. et al., 2020; Weiler et al., 2025) are responsible for triggering mental fatigue. Some sport-specific demands frequently mentioned by elite athletes were inhibition (e.g., suppressing emotions after a controversial referee call), or being asked to continuously make decisions during a game (e.g., passing vs. shooting in soccer). In particular, the insights into sport-specific demands as antecedents to mental fatigue have been useful to inform more sport-specific study designs to investigate mental fatigue. For instance, Coutinho et al. (2017) asked participants in a first task to perform a 20-min whole-body coordination task that involved performing different general exercises in the ladder drill (e.g., jumping, running backwards etc.) while juggling a tennis ball to increase the mental demands of the task. In line with the authors' assumptions, participants reported higher levels of mental fatigue and displayed lower physical and tactical performance in a second task (a small-sided game). In other studies, mental fatigue was induced by asking junior soccer players to solve film-based tactical tasks for 30 min (Ciocca et al., 2022) or by increasing the mental demands of an established pre-match routine through smartphone use (Greco et al., 2017). These tasks led to increased sensations of mental fatigue and impaired subsequent motor performance. Bian et al. (2022) utilized a 20-min repeated interval Loughborough Soccer Passing Test (LSPT) as a soccer-specific motor task to induce mental fatigue. The task represents some of the mental (e.g., decision-making) and soccer-specific demands (e.g., passing, dribbling, control) soccer players are frequently confronted with during play (see also Kunrath et al., 2020a; Gantois et al., 2020; Thompson et al., 2019). In this task, participants must follow verbal instructions regarding a random passing order 16 times and perform soccer-specific skills as quickly and as accurately as possible.

Another promising venue for inducing mental fatigue in a more ecologically valid but also highly standardized setting is to adopt modern technology in soccer (Ehmann et al., 2021, 2022). One of the most established research tools in soccer is the Footbonaut (Musculus et al., 2022), which is primarily used for soccer-specific diagnostics as well as training purposes (Beavan et al., 2018; Saal et al., 2018). In the standardized version of this task, the participants receive balls from a ball-passing machine and are asked to control them and pass them to highlighted targets within the Footbonaut (for the setup of the Footbonaut, see the Methods Section). Due to the soccer-specific mental demands of the Footbonaut, it also appears to challenge specific movement execution and cognitive functions for a prolonged period, therefore mimicking the receiving and passing in real-world soccer better than other approaches and providing a more ecologically valid research setting. Therefore, the aim of this study is to develop and validate a mentally demanding, sport-specific task in the Footbonaut to experimentally induce mental fatigue and to investigate its effects on subsequent cognitive and soccer-specific performance.

## 2 Methods

### 2.1 Participants

An *a priori* power analysis revealed that a sample size of 24 soccer players was required to find a moderate effect (Brown et al., 2019; analysis: within-subjects analysis of variance; parameters: *f* = 0.30, α = 0.05, 1 – β = 0.80, *r*_repeated measures_ = 0.50, ε = 1; Faul et al., 2009). Due to potential dropouts, a total of 30 adult male soccer players (age: *M* = 24.6 ± 4.5 years) were recruited, with three players being excluded due to illness or injury during the testing period. The participants were well-trained, competitive soccer players from the sixth German football league and included 11 defenders, 13 midfielders and 3 forwards. Goalkeepers were excluded from the study due to the different position-specific requirements (Sarmento et al., 2024). All players were free of (chronic) diseases, injuries and color vision deficiencies. The players were recruited and contacted via their local soccer teams. Participation in the study was voluntary and could be withdrawn at any time without any consequences and without specifying any reasons. Eligible players signed informed consent forms describing the study procedure and outlining the potential risks of the study. The ethical integrity of the study and all its procedures were approved by the local ethics committee (November 2023, Approval number 2023-51), and the study was conducted in accordance with the Helsinki declaration.

### 2.2 Study design and procedures

**Figure 1:**
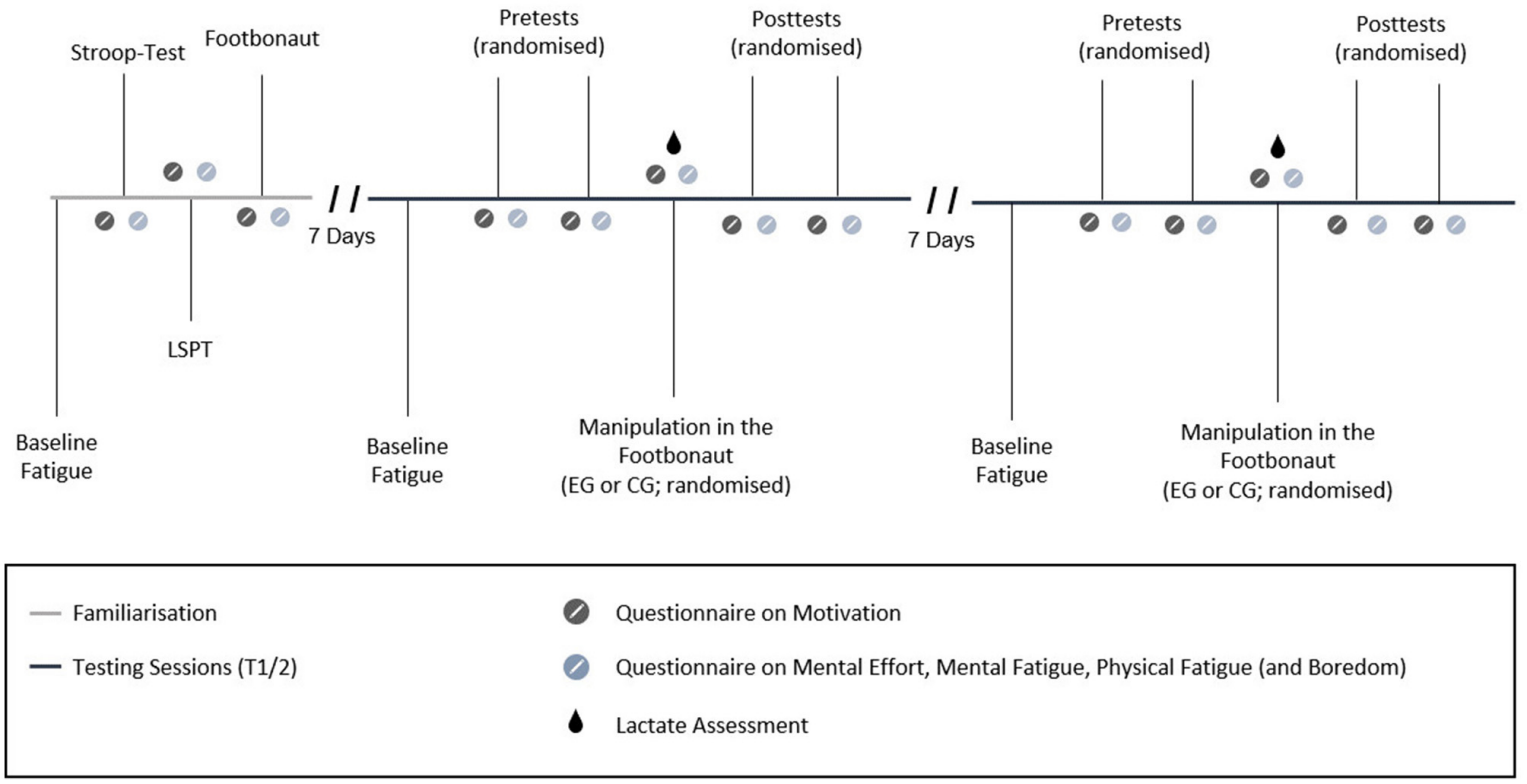
Timeline of testing sessions (T2 and T3).

A randomized, counter-balanced experimental within-subjects design was employed, consisting of three times of measurement with a 7-day interval between each visit (for a graphical depiction of the procedure, see Figure 1). The first time of measurement (T1) served as a familiarization visit, as players were exposed to all testing and assessment procedures in order to reduce potential learning effects at the two subsequent times of measurement (i.e., T2 and T3). At T1, the players were further familiarized with the concepts of mental and physical fatigue by being provided with a standardized definition (Russell et al., 2022). Each visit took place at the same time of the day, to minimize the potential impact of circadian rhythms.

T2 and T3 were performed by the players in a randomized and counterbalanced order (randomization.com). At T2 and T3, players followed the subsequent procedures: (1) a questionnaire on their health status; (2) self-assessments regarding their motivation, mental effort, mental fatigue, physical fatigue and boredom (via Visual Analog Scales (VAS); for this procedure, see Bian et al., 2022); (3) a series of pretests for assessing the dependent variables including a short version of the Stroop task (i.e., variable 1; see Pageaux et al., 2015) and three trials of the LSPT (i.e., variable 2; see Bian et al., 2022); (4) a 30-min passing task in the Footbonaut consisting of four sets of 80 balls, which was used to experimentally manipulate mental fatigue (i.e., independent variable: mental fatigue: yes vs. no); and (5) several post-tests (consisting of the same tasks as performed in the pretest; for a detailed description of each measure and treatment, see below). During T2 and T3, additional physiological parameters [heart rate (HR), blood lactate concentration (Bla)] were measured. At T3, a brief demographic questionnaire was included as well as a trait questionnaire to record the players' level of trait self-control (SCS-K-D; Bertrams and Dickhäuser, 2009). The latter is not relevant for the current study and is therefore not described in more detail. After completing the final questionnaires, the players were debriefed and the experimenters thanked them for participating.

### 2.3 Control measures

All subjective parameters were measured by using an online survey tool (Unipark, QuestBack 13 GmbH, Köln, Germany).

Player health status was assessed at each visit with the German version of the Physical Activity Readiness Questionnaire (PARQ; Thomas et al., 1992). The scale consists of seven dichotomous items (e.g., “Have you experienced any sort of chest pain during the last month?”, yes vs. no). Players would have been excluded had they reported any physical symptoms, which was not the case in the present study.

The following five subjective parameters were measured via VAS (for this approach, see also Van Cutsem and Marcora, 2021; Kunrath et al., 2020a; Thompson C. et al., 2020): (1) motivation (“Please indicate how motivated you are regarding the upcoming task.”), (2) mental fatigue (“Please indicate the level of your current state of mental fatigue.”), (3) physical fatigue (“Please indicate the level of your current state of physical fatigue.”), (4) mental effort (“Please indicate your current state of perceived mental effort.”) and (5) boredom (“Please indicate how bored you currently feel.”). The VAS referred to the Stroop task, the LSPT and the Footbonaut: while motivation was assessed before the respective upcoming task, mental fatigue, physical fatigue, mental effort and boredom were assessed after the respective task was completed. Further, mental and physical fatigue were also assessed at the beginning of T2 and T3 to determine the level of baseline fatigue. The VAS is a 100 mm horizontal line, ranging from 0 (“not at all”) to 100 (“maximum”), and players were instructed to indicate the level to which each attribute applied to them in the given moment by placing a vertical mark on the line at the point that best reflects how they felt.

Physiological assessments included analyses of the HR and the Bla. The HR was monitored by using an HR chest strap (Polar H10 Sensor, Kempele, Finland). To determine the Bla, capillary blood samples from the hyperaemised earlobe were taken (Biosen C-Line Sport, EKF-diagnostic GmbH, Barleben, Germany). Both physiological parameters were assessed five times in total for each player at T2 and T3, immediately before performing the Footbonaut task (baseline values) and after each set of 80 balls played. These measures were taken to avoid the interference of uneven physical demands between the two versions of the Footbonaut.

### 2.4 Mental fatigue manipulation

**Figure 2:**
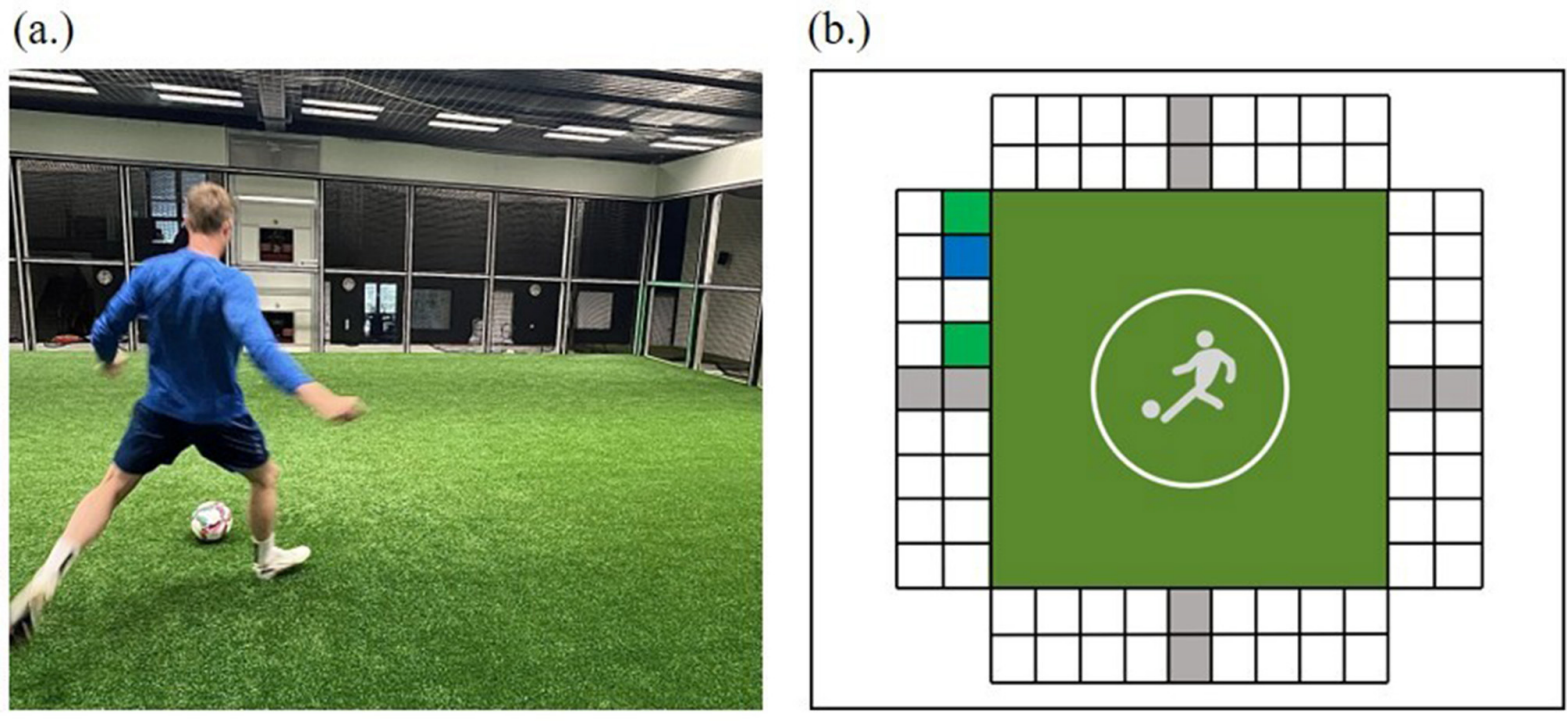
**(a)** Photograph of the Footbonaut in use, showing a player interacting with the system during a training session. **(b)** Schematic representation of the experimental condition in the Footbonaut.

Mental fatigue was experimentally manipulated (Mental fatigue: yes vs. no) with a soccer passing task in the Footbonaut in a counterbalanced order. The Footbonaut (C-Goal GmbH, Berlin, Germany) is a well-established training and research tool for soccer players (see also Beavan et al., 2018; Saal et al., 2018). The Footbonaut consists of a 14 m^2^ artificial turf pitch surrounded by four grid walls (see Figure 2). Each wall consists of 18 panels arranged in two rows, an upper row (nine panels) and a lower row (nine panels). In the middle of each row a ball canon is placed, which is used to pass the balls toward the middle of the Footbonaut. In total, eight ball canons are located inside the Footbonaut. The panels are surrounded by a light-emitting diode to show the players which field is currently active or should be targeted. All panels contain a light barrier, which has been utilized to record the players' passing performance (accuracy) and processing speed (response time).

In the current study, two different soccer passing tasks in the Footbonaut were used to manipulate mental fatigue: a mentally less demanding standard training session as the control condition, and an adapted mentally more demanding training session as the experimental condition. In both conditions, the task lasted ~30 min (for more information regarding the recommended durations of mentally demanding tasks, see also Van Cutsem et al., 2017) and consisted of 320 trials, which were divided into four sets of 80 balls each. Between each set, there was a break of ~30 s, which was used to assess physiological parameters (i.e., Bla, HR; see above for more information). In both conditions, only the lower ball canons and the lower target fields were included (i.e., a total of four ball canons and 36 target fields were active). While both conditions followed the same framework, they differed in terms of their complexity and mental demands.

**Figure 3:**
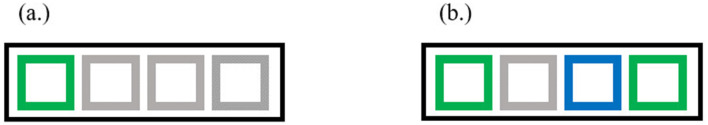
Detailed schematic representation of the **(a)** standard and **(b)** experimental task in the Footbonaut.

In both conditions, the players positioned themselves in the middle of the artificial turf pitch and the experimenter started the session. After a 10-s countdown, the program started. The direction of the incoming balls was always announced by an audio-visual signal (sound plus red light) from the ball cannon. The ball was played by the ball cannon with a delay of 800 ms after the signal. At the same time, a visual signal (green light) followed, by illuminating one of the panels. In the control condition, we used the standard training session (the mentally less demanding; see Figure 3), as the player's task was to receive the incoming ball with a first contact and shoot it as quickly as possible into the illuminated panel with a second contact. Immediately after the ball passed the light barrier, the ball canon emitted another audio-visual signal to indicate the direction of the subsequent ball. The player's task was the same across all four series. The players were instructed to process the incoming balls as accurately and quickly as possible.

In the experimental condition, players were also instructed to perform the soccer passing task in the Footbonaut. However, the instructions were more difficult to follow than in the control condition and thus the task required more mental effort than in the control condition. Instead of one target field, three target fields were activated for each trial. Two of the target fields lit up in the same color (green) and one in a different color (blue; see Figure 3). The instructions as to which field was the target field varied across the four series in order to keep the task difficulty at a high level over the entire course of the 30-min task, as well as to avoid sensations of boredom or lower motivation (for a discussion, see Wolff et al., 2022; for the exact instructions, see Supplementary material ESM 1). As in the control condition, the players were instructed to process the incoming balls as accurately and quickly as possible.

### 2.5 Dependent variables

#### 2.5.1 Stroop task

In order to test the effects of this mental fatigue manipulation on cognitive performance, players worked on a 3-min version of the Stroop task using OpenSesame (version 4.0.5.). The Stroop task is a well-established task which has been frequently applied in previous lab-based research on mental fatigue (Van Cutsem et al., 2017; Badin et al., 2016; Pageaux et al., 2014; Smith et al., 2016). It primarily assesses inhibitory control, a specific facet of cognitive performance.

In the current study, each Stroop task consisted of a series of incongruent color words (red, blue, green and yellow) which were repeatedly displayed in different font colors (either red, blue, green and yellow) on a computer screen with a black background. The players were instructed to name each word's font color rather than the semantic meaning of the word as quickly and accurately as possible by pressing the respective color-masked button on the keyboard (e.g., if the word yellow appears in blue, the button “blue” is to be pressed). This is the case except when the word “red” appeared: then, according to an extra rule, the players should no longer respond to the font color but to the semantic meaning of the word (e.g, if the word red appears in blue, the button “red” is to be pressed) (Pageaux et al., 2015). The stimulus faded from the screen as soon as the players responded, followed by a new stimulus that appeared immediately. The words presented and their font colors were randomly selected (100% incongruent). Players' performance was assessed in terms of the total number of trials completed, their errors and response latencies.

#### 2.5.2 Loughborough Soccer Passing Test

**Figure 4:**
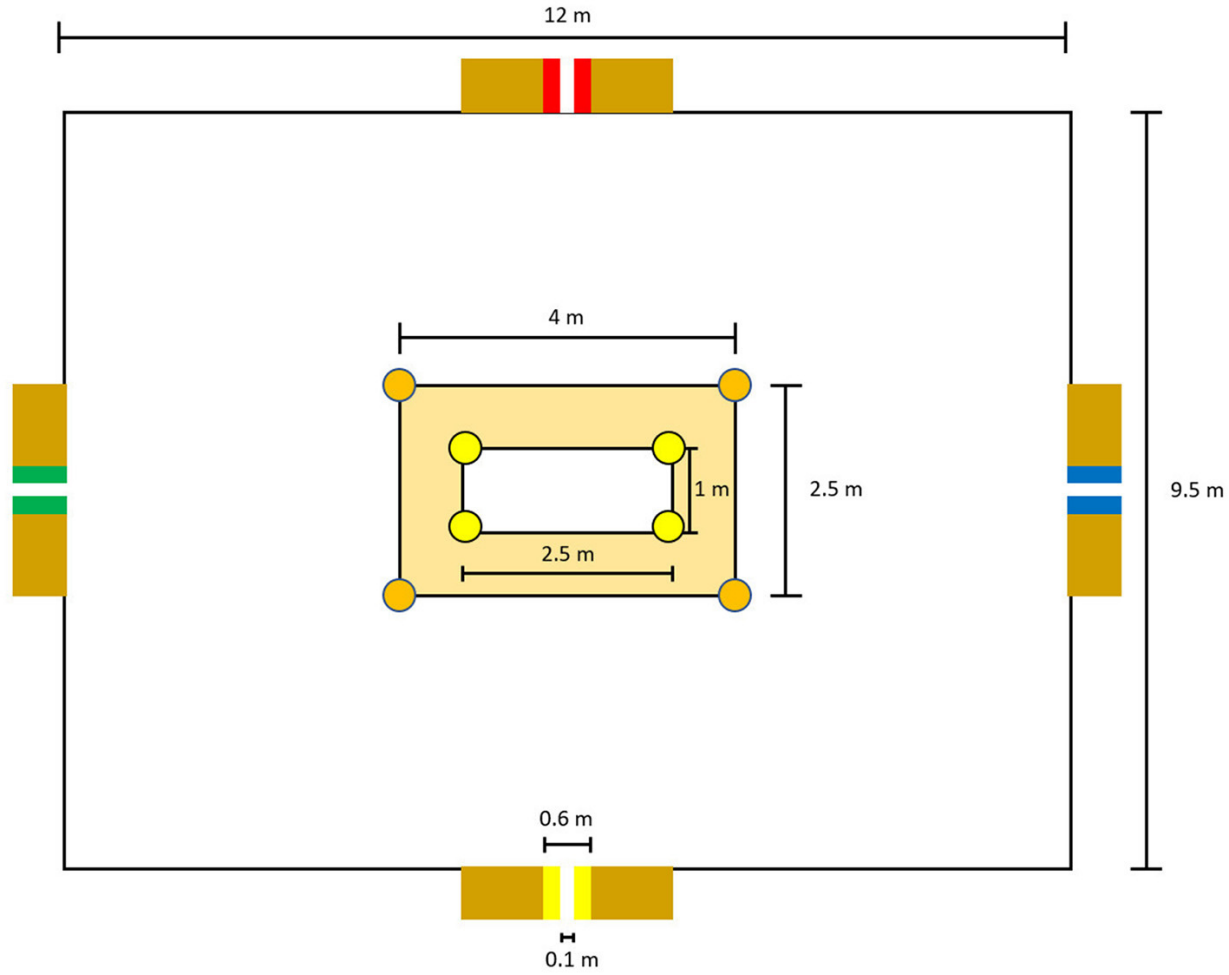
Schematic representation of the LSPT.

As a second, more sport-specific outcome, players performed three sets of the LSPT. The LSPT as a soccer-specific task has also been applied in previous research on mental fatigue (Bian et al., 2022; Grgic et al., 2022; Smith et al., 2016). Figure 4 illustrates the setup of the LSPT, which consists of three main components: central zone, passing zone and four colored passing targets (red, blue, green, and yellow), which surround the rectangular playing area (9.5 × 12 m). These four colored passing targets were attached on rebound boards (standard benches) using colored tape (0.25 × 0.6 m). Additionally, a white stripe (0.25 × 0.1 m) was vertically taped in the middle of the passing targets. To distinguish the different zones inside the playing area, colored cones were used.

Each LSPT set consisted of 16 passes, as players were instructed to always pass the ball as quickly and accurately as possible toward the target color, which was called out by the experimenter (Grgic et al., 2022; Smith et al., 2016). The order of the targets was randomized (radomization.com) and each set included eight short (red, yellow) and eight long passes (green, blue). Between the sets, there was a break of 75 s. Players started with the ball in the middle of the central zone. After the experimenter named the first target color, the movement time until completion of the 16 passes (in milliseconds) was measured. The time was stopped when the ball on the final pass hit the target area. Depending on how accurately the task was completed, the players were additionally able to improve or worsen their times. This was done on the basis of bonus times (e.g., hitting the white stripe within the colored target area) or time penalties [e.g., missing the bench completely or playing the incorrect bench (+5 s)], which were outlined before the start of the test (for more information, see Supplemental Material ESM 1). The task was recorded on video from two perspectives, and the individual performance was rated afterwards by an independent rater. Players' performance was assessed in terms of the errors during the test (penalty time), the movement time to complete the test (movement time) and the movement time plus the penalty time (performance time). For the statistical analysis, sum scores consisting of the single scores of the three trials were calculated for each performance parameter.

#### 2.5.3 Statistical analyses

All statistical analyses were conducted using JASP, version 0.18.3 (Amsterdam, Netherlands). We used a significance level of *p* = 0.05. Effect sizes were interpreted according to Cohen (1988). To increase comprehensibility, we present the statistical hypothesis tests used directly in the Results Section. In several places, two-way repeated measures ANOVAs were used. For all of these ANOVAs, only the interaction effects relevant to the hypotheses are reported. All associated main effects are reported in the Supplementary Material ESM 3; Tables S8–S13. Data sphericity was verified by Mauchly's test, and the Greenhouse Geisser correction was applied when this assumption was violated. In case of significant interactions, we used subsequent Bonferroni corrected *post hoc* tests.

## 3 Results

### 3.1 Preliminary analyses

To test potential pre-experimental differences in mental and physical fatigue between the two conditions, paired *t*-tests (Condition: experimental vs. control) were carried out. Mental fatigue before starting the experiment at T2 and T3 did not differ between the experimental (*M* = 16.07; *SD* = 15.41) and the control condition (*M* = 18.26; *SD* = 20.00), *t*_(26)_ = 0.63, *p* = 0.535, *d* = 0.12. The same was true for physical fatigue (experimental condition: *M* = 18.85; *SD* = 21.62; control condition: *M* = 19.89; *SD* = 17.82), *t*_(26)_ = 0.29, *p* = 0.778, *d* = 0.06.

To analyze the data for potential order effects, we conducted paired *t*-tests and two-way repeated measures ANOVAs. We found no order effects regarding the (performance-related) dependent variables (see Supplementary ESM 2; Tables S1–S7 for detailed results concerning order effects).

### 3.2 Manipulation checks

We assumed the manipulation of mental fatigue to be successful if the experimental condition in the Footbonaut elicited higher perceptions of mental effort and mental fatigue compared to the cognitively less demanding task in the control condition on a subjective level. Accordingly, we used paired *t*-tests (condition: experimental vs. control) to test whether mental effort and mental fatigue as well as the other subjective control measures differed between the two conditions (see Table 1). Contrary to our *a priori* assumptions, players reported higher levels of mental fatigue in the control condition compared to the experimental condition. The same applied for the self-reported level of physical fatigue. No statistically significant differences were found between the conditions for mental effort and boredom. Further, there were no condition-specific differences in the self-reported levels of motivation of the players regarding the upcoming Footbonaut task.

**Table 1:** Differences in the subjective control measures between the experimental and the control condition in the Footbonaut task (motivation assessed before, all other measures assessed after performing the task).

**Subjective measures**	**Experimental condition**		**Control Condition**		***t*_(26)_**	***p***	***d***
	***M***	***SD***	***M***	***SD***			
Motivation	89.67	11.33	90.19	11.33	0.44	0.663	0.09
Mental effort	59.59	19.12	62.70	19.94	0.95	0.350	0.18
Mental fatigue	51.00	18.63	58.59	22.52	2.25	0.033	0.43
Physical fatigue	60.48	21.25	68.37	17.62	2.20	0.037	0.42
Boredom	15.41	24.63	18.26	25.89	0.50	0.621	0.10

Additionally, we expected a lower Footbonaut performance in the experimental condition compared to the control condition (i.e., lower accuracy and longer response times) due to the higher complexity of the instructions in the experimental condition. We used two way-repeated measures ANOVAs [Condition (experimental vs. control) × Sets in the Footbonaut (1 vs. 2 vs. 3 vs. 4)] to test these additional assumptions. Descriptive statistics regarding the performance in the Footbonaut are displayed in the Supplementary Material ESM 3; Table S8.

The results suggest that the mental fatigue manipulation was successful, as players' performance in the Footbonaut task was significantly lower in the experimental as opposed to in the control condition (i.e., lower accuracy scores) over the course of the sets, *F*_(3, 78)_ = 7.42, *p* < 0.001, $\eta_{p}^{2}$ = 0.222 (for a graphical representation, see Supplementary ESM 3; Supplementary Figure S1). The two conditions did not differ in their average response times over the course of the sets, *F*_(1.81, 47.15)_ = 1.46, *p* < 0.243, $\eta_{p}^{2}$ = 0.053.

### 3.3 Control measures

All descriptive statistics and additional information regarding the main effects for the following analyses of the control measures are displayed in the Supplementary Material ESM 3; Supplementary Tables S9–S11.

#### 3.3.1 Subjective measures

Regarding the subjective control measures, it was assumed that in the experimental condition, the Stroop task and the LSPT would be perceived as more effortful und more fatiguing than in the control condition. Further, a lower motivation was expected for the experimental condition. To test this, two-way repeated measures ANOVAs [Condition (experimental vs. control) × Time of Assessment (pre vs. post)] were performed for motivation, mental effort, mental fatigue and physical fatigue.

Regarding the Stroop test, there were no significant differences in the change of mental effort from pre- to post-test between the experimental and the control condition, *F*_(1, 26)_ = 0.01, *p* = 0.945, $\eta_{p}^{2}$ < 0.001. The same was true for mental fatigue, *F*_(1, 26)_ = 0.20, *p* = 0.662, $\eta_{p}^{2}$ = 0.007, and physical fatigue, *F*_(1, 26)_ = 0.34, *p* = 0.567, $\eta_{p}^{2}$ = 0.013. There were also no significantly different changes between the experimental and the control condition in mental effort, *F*_(1, 26)_ = 1.14, *p* = 0.295, $\eta_{p}^{2}$ = 0.042, mental fatigue, *F*_(1, 26)_ = 0.10, *p* = 0.752, $\eta_{p}^{2}$ = 0.004, and physical fatigue, *F*_(1, 26)_ = 0.00, *p* = 0.968, $\eta_{p}^{2}$ < 0.001, regarding the LSPT.

Further, motivation did not change significantly from pre- to post-test in the experimental compared to the control condition in terms of the Stroop task, *F*_(1, 26)_ = 1.51, *p* = 0.230, $\eta_{p}^{2}$ = 0.055, and the LSPT, *F*_(1, 26)_ = 0.17, *p* = 0.685, $\eta_{p}^{2}$ = 0.006.

#### 3.3.2 Physiological measures

Bla and HR were assumed to not change significantly in the course of both conditions due to the same physiological demands. Therefore, we performed two-way repeated measures ANOVAS [Condition (experimental vs. control) × Assessments in the Footbonaut (1 vs. 2 vs. 3 vs. 4 vs. 5)] for these physiological measures.

There were no significant interaction effects for Bla between the conditions and the sets of balls passed, *F*_(2.17, 56.43)_ = 0.24, *p* = 0.806, $\eta_{p}^{2}$ = 0.009. Likewise, HR did not change differently between the experimental and the control condition over the course of the sets in the Footbonaut, *F*_(1.58, 40.95)_ = 0.15, *p* = 0.809, $\eta_{p}^{2}$ = 0.006.

### 3.4 Main analyses

Based on the findings of previous studies that mentally more demanding tasks increase sensations of mental fatigue and impair subsequent performance compared to less mentally demanding tasks (e.g., Brown et al., 2019), it was hypothesized that performance would be impaired after the experimental manipulation in the Stroop task (less trials, more errors, longer response times) and in the LSPT (longer penalty times, longer movement times, longer performance times) compared to after the control condition. To test these hypotheses, we conducted two-way repeated measures ANOVAs [Condition (experimental vs. control) × Time of Assessment (pre vs. post)] for each performance indicator in the Stroop task and the LSPT, respectively.

#### 3.4.1 Cognitive performance—Stroop task

Descriptive statistics and additional information regarding the main effects for the Stroop task parameters can be found in the Supplementary Material ESM 3; Table S12. In contrast to our hypothesis, the interaction between condition and performance change in the Stroop task was not statistically significant for the number of trials completed, *F*_(1, 26)_ = 2.01, *p* = 0.168, $\eta_{p}^{2}$ = 0.072, the errors, *F*_(1, 26)_ = 1.64, *p* = 0.211, $\eta_{p}^{2}$ = 0.059, and the response time, *F*_(1, 26)_ = 0.58, *p* = 0.452, $\eta_{p}^{2}$ = 0.022. Accordingly, performance in the Stroop task did not change significantly from pre- to post-test in the experimental compared to the control condition.

#### 3.4.2. Soccer-specific performance—LSPT

**Figure 5:**
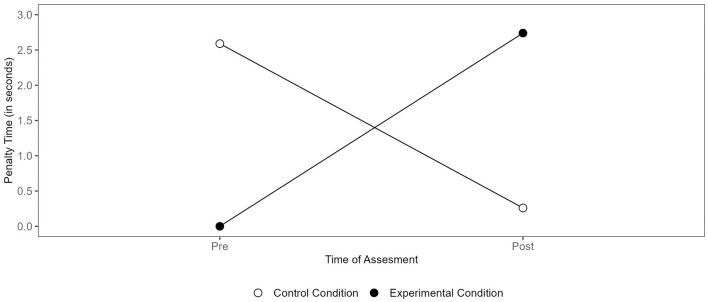
Condition-specific differences in the penalty time (LSPT).

Descriptive statistics and additional information regarding the main effects for the LSPT parameters can be found in the Supplementary Material ESM 3; Table S13. Contrary to our expectations, no interaction effect was found between condition and change in required movement time to complete the LSPT from pre- to post-test, *F*_(1, 26)_ = 0.94, *p* = 0.342, $\eta_{p}^{2}$ = 0.035. As predicted, an interaction effect was identified between the condition and changes in penalty time from pre- to post-test, *F*_(1, 26)_ = 4.36, *p* = 0.047, $\eta_{p}^{2}$ = 0.143. However, *post-hoc* analyses did not reveal any significant differences. Descriptively, a performance improvement from pre- to post-test (i.e., a decrease in penalty times) was shown in the control condition, while performance losses from pre- to post-test (i.e., an increase in penalty times) were found in the experimental condition (see Figure 5).

**Figure 6:**
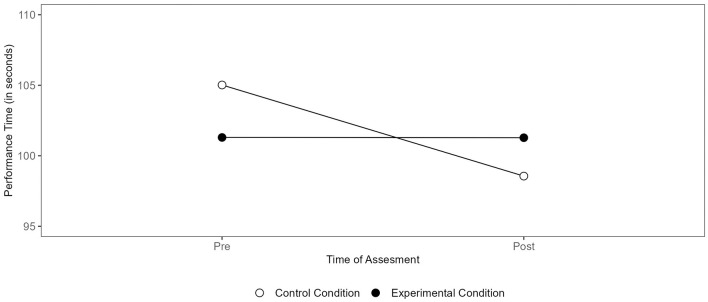
Condition-specific differences in performance time (LSPT).

The interaction between condition and changes from pre- to post-test in performance time was significant as well, *F*_(1, 26)_ = 7.79, *p* = 0.010, $\eta_{p}^{2}$ = 0.230. *Post hoc* analyses revealed that, in the experimental condition, no changes in performance time from pre- to post-test were detected (*p* > 0.999), while in the control condition, players' performance time was significantly better after the Footbonaut task than before (*p* = 0.013; see Figure 6).

## 4 Discussion

The aim of the current study was to increase the ecological validity in mental fatigue research by developing a soccer-specific manipulation task to induce mental fatigue. Based on the assumption that higher mental effort leads to higher levels of mental fatigue (e.g., Van Cutsem and Marcora, 2021), we established two different experimental conditions for a Footbonaut task that differed from each other in the amount of mental effort required to follow the respective instructions (mentally more demanding experimental condition vs. mentally less demanding control condition; order counterbalanced) and investigated their effects on subsequent performance (i.e., a mentally demanding cognitive Stroop task and a mentally demanding soccer-specific LSPT). It was assumed that participants would perform worse in the experimental condition in both tasks compared to the control condition. Similar to the mentally demanding version of the well-established Stroop task used to induce mental fatigue (i.e., primarily incongruent color words; e.g., Pageaux et al., 2015), the instructions for the Footbonaut task in the experimental condition were more complex than in the control condition as they contained additional mental demands that play a crucial role in soccer (i.e., inhibition, decision making; e.g., Thompson et al., 2019).

To test whether the experimental manipulation was successful, subjective and performance-related manipulation checks were conducted following the Footbonaut task. While on a subjective level, surprisingly, players reported higher levels of mental and physical fatigue in the control condition, in line with our expectations, players performed significantly worse in the experimental condition (i.e., lower passing accuracy) in the Footbonaut task. On an objective level, this indicates that the mental demands were higher in the experimental condition than in the control condition. As expected, no differences between the two conditions were found in terms of motivation, mental effort or boredom. Furthermore, there were no statistically significant differences in physical fatigue, as the Bla and HR scores did not differ between the two experimental conditions. Contrary to our expectations, there were no performance-related differences between the two conditions in the Stroop task (i.e., the total number of trials completed, the errors, and the response time). However, as predicted, the interaction patterns in the soccer-specific LSPT are consistent with a negative impact of the experimental condition on performance. Although *post-hoc* comparisons for penalty times were not statistically significant, the interaction effect points toward increased penalties under the experimental condition. The performance time interaction was primarily driven by improvements in the control group, whereas participants in the experimental condition showed no such gains—indicative of a performance stagnation aligned with the hypothesized impairment. The effect sizes observed for changes in LSPT performance (η_*p*_^2^ = 0.143 and 0.230, respectively) reflect strong interaction effects. These values exceed the small-to-medium effects of non-sport-specific cognitive exertion tasks on subsequent physical performance found in meta-analytical research (*g* = −0.38, corresponding to a η_*p*_^2^ of around 0.035; Brown et al., 2019). However, given the substantial differences in task specificity and experimental design, such comparisons should be interpreted with caution.

The findings of the current study deliver initial evidence that the Footbonaut task appears to be a sport-specific and thus ecologically valid task to experimentally manipulate mental fatigue. However, there are several limitations which need to be discussed in order to further improve the validity of the Footbonaut task.

### 4.1 Limitations and future directions

It needs to be critically mentioned that the findings regarding several subjective parameters, which were measured via VAS, showed certain inconsistencies between the two conditions. Although the VAS is a frequently applied introspective measure in mental fatigue research, the assessment of mental fatigue as a psychobiological state is considered to be challenging (Van Cutsem and Marcora, 2021). The subjective assessments of mental fatigue and related parameters (e.g., mental effort) by using introspective self-assessments have been repeatedly criticized, as they seem to be prone to self-report biases (Bian et al., 2022; Thompson et al., 2019). It might be reasonable to assume that participants with relatively high or low levels of mental fatigue tend to over- or underestimate their current state (Thompson et al., 2019). Several mental fatigue researchers argue that such difficulties are partly attributable to a lack of understanding of what mental fatigue actually is, for instance by confusing mental and physical fatigue (e.g., Bian et al., 2022; Thompson et al., 2019). These methodological factors might have contributed to the aforementioned inconsistencies in the current study, although participants were familiarized in detail with both concepts (Russell et al., 2022) at the first time of measurement. Accordingly, the current results in terms of the subjective assessments by VAS should be considered carefully and not in isolation, but rather in context with the more representative objective results (i.e., performance differences between the two experimental conditions).

Another issue to be discussed is the fact that there was only a detrimental effect of mental fatigue on performance in the LSPT but not in the Stroop task. In this context, it should be noted that although the Stroop task is one of the most established tasks in mental fatigue research, it has been repeatedly criticized for its low level of ecological validity for research in the field of sport and exercise psychology (Bian et al., 2022; Thompson et al., 2019). One explanation of why there was only an effect of mental fatigue on soccer-specific performance is provided by the current body of studies in related fields of research, particularly that of (perceptual-)cognitive training (e.g., Ehmann et al., 2022; Zentgraf et al., 2017). In this field, researchers investigate the effects of training a certain perceptual-cognitive skill (e.g., executive functions) on subsequent performance at different levels: (1) task-specific (i.e., the extent of performance improvement in the identical task), (2) near transfer (i.e., the extent of performance improvement in tasks which require the same perceptual-cognitive skills) and (3) far transfer (i.e., the extent of performance improvement in unrelated cognitive tasks; for an overview, see also Fleddermann et al., 2019; Zentgraf et al., 2017). There is a large body of supporting evidence for task-specific as well as near transfer effects of perceptual-cognitive training (e.g., Fransen, 2022; Gobet and Sala, 2023). However, and to the best of our knowledge, there is only a limited amount of research on the effects of perceptual-cognitive training on sports performance (Fransen, 2022; Gobet and Sala, 2023; Scharfen and Memmert, 2021). The findings of the current study appear to blend rather seamlessly into research on perceptual-cognitive training, as we only found an effect of the sport-specific mentally demanding Footbonaut task on another sport-specific task which was also mentally demanding, similar to a near transfer effect. The Footbonaut task in the control condition appears to be more similar to the requirements of the LSPT than the Footbonaut task in the experimental condition. This could have contributed to a near transfer training effect and may explain why, at least descriptively, improvements in the LSPT are evident in the control condition but not in the experimental condition. In contrast, there was no evidence of a far transfer effect of the Footbonaut task, as cognitive performance (i.e., Stroop performance) did not differ significantly between the two experimental conditions. Future studies should dig deeper into whether near vs. far transfer effects can also be found in mental fatigue. Such research could provide a scientific basis for the development of effective manipulation tasks in order to investigate the real-life effects of mental fatigue.

In this context, the validity of the control condition also warrants further consideration. Although it was designed as a cognitively less demanding baseline condition, the players reported higher levels of mental fatigue following the control task compared to the experimental task even though boredom ratings were not statistically different between the two conditions. It is plausible that the cognitive load imposed by the control task was not sufficiently distinct from that of the experimental condition, given the similarity in task requirements which calls into question the ecological validity of the manipulation. These findings underscore the importance of interpreting control conditions not only in terms of cognitive load level, but also with regard to task characteristics that influence subjective awareness of mental exertion. Nonetheless, the significant performance disparities observed suggest that the experimental condition did elicit higher mental and cognitive demands. This research represents an initial step toward increasing ecological validity, aiming to provide insights that inform subsequent investigations in mental fatigue research. Future studies should consider developing a control condition that minimizes cognitive engagement to better isolate the effects of mental fatigue.

The Footbonaut task had a detrimental effect on technical abilities in the LSPT (i.e., penalty and performance time), but not on the time required to perform the LSPT (i.e., movement time). These results are in line with previous study results (Bian et al., 2022; Filipas et al., 2020; Smith et al., 2016). Past studies have shown that the state of mental fatigue favors a trade-off between speed (e.g., in the form of movement time) and accuracy (e.g., in the form of penalty time; Sun et al., 2021). Accordingly, it is likely that losses in accuracy are accepted in order to maintain performance in terms of (processing) speed. In mental fatigue research, this form of compensation mechanism is explained using Smith's conceptual model (Smith et al., 2018). According to this model, it can be assumed that mentally demanding tasks activate the anterior cingulare cortex, leading to an increased occurrence of adenosine and a reduced release of dopamine (Lorist et al., 2005; Smith et al., 2018). Such changes may further result in the impairment of several executive functions, including attentional allocation. Due to the limited attentional resources, players would still be able to perform subsequent tasks quickly, but no longer accurately. Further, it demonstrates the importance of including biological manipulation checks (e.g., electroencephalography) alongside subjective ones in future research in order to respect the multidimensional nature of mental fatigue (Habay et al., 2021).

Further, although the protocol was successful to some extent in inducing mental fatigue in football players, the effect on football-specific performance was limited (e.g., small effect size) and there was no evidence regarding cognitive performance. In future studies, this problem could be addressed in several ways. One possibility to enhance the effectiveness of mental fatigue manipulations in future research is to increase the cognitive complexity and contextual interference of the applied tasks (Bian et al., 2022; Habay et al., 2021). For example, frequent task switching could help prevent proceduralization, which refers to the automatization of task execution over time, often leading to a decline in mental demands and thus a reduced likelihood of inducing mental fatigue (e.g., Boksem et al., 2005). Furthermore, extending the task duration beyond 30 min might be necessary, particularly when working with highly skilled athletes who are accustomed to sustaining mental demands over longer periods (Boat et al., 2020; Ciocca et al., 2022; Soylu et al., 2021). Complementary to this, future studies might consider incorporating multisensory distractors—such as combining visual cues with auditory stimuli like crowd noise—which may further increase task complexity and cognitive load (Ferreira et al., 2024). At the same time, the design of the present study—with alternating task types and short breaks between task blocks—was not chosen arbitrarily. Rather, it mirrors the intermittent nature of soccer-specific cognitive demands, where players are repeatedly required to switch between different mental operations under time constraints. Although alternating-type designs and interspersed recovery periods have been shown to reduce the onset of mental fatigue under certain conditions (Van Cutsem and Marcora, 2021; Weiler et al., 2025), they may also help to maintain cognitive engagement and prevent boredom—particularly in applied sport settings. This aligns with the design of many established mental fatigue paradigms, such as the Stroop task, which also feature repeated blocks separated by brief breaks (Dallaway et al., 2022). Thus, the balance between cognitive challenge and ecological validity remains a crucial aspect in future research aiming to simulate soccer-specific mental demands.

It is important to acknowledge that our study's sample consisted exclusively of semi-professional male soccer players. This specificity limits the generalizability of our findings to elite athletes and other sports contexts. Furthermore, the absence of female participants precludes conclusions about the effects of the Footbonaut task on female soccer players (for a more detailed discussion on the gender—data gap in sports, see also Curran et al., 2019).

### 4.2 Conclusion

Our findings imply that cognitive-motor interference induced by 30-min Footbonaut technology-based training (characterized by decision-making demanded passing/shooting tasks) may induce mental fatigue in soccer players. While no effects on cognitive performance in the Stroop task were detected, detrimental effects of induced mental fatigue on soccer-specific performance in the LSPT were revealed. In particular, a deterioration in the penalties and the performance time compared to the control condition occurred. Induced mental fatigue could disrupt the accurate and fast execution of soccer-specific skills, thereby attenuating the near-transfer benefits seen after repetitive motor practice with less mental demands (as in the control condition). Overall, the experimental manipulation of mental fatigue via the Footbonaut could be a useful alternative for inducing and investigating mental fatigue in soccer in a more ecologically valid way, even if our study is only a first step.

## Data Availability

The raw data supporting the conclusions of this article will be made available by the authors, without undue reservation.
