# Supplementary material for: Increasing ecological validity in mental fatigue research—A Footbonaut study
Source: Front Psychol. 2025 May 27;16:1586944. doi: 10.3389/fpsyg.2025.1586944 (PMC12149105; doi:10.3389/fpsyg.2025.1586944)
Supplement: Supplementary file 1 [file Data_Sheet_1.pdf]

Abbreviations

Bla = Blood lactate concentration

HR = Heart rate

LSPT = Loughborough Soccer Passing Test

T = Time of measurement

VAS = Visual Analog Scale
