# Supplementary material for: Increasing ecological validity in mental fatigue research—A Footbonaut study
Source: Front Psychol. 2025 May 27;16:1586944. doi: 10.3389/fpsyg.2025.1586944 (PMC12149105; doi:10.3389/fpsyg.2025.1586944)
Supplement: Supplementary file 3 [file Data_Sheet_3.pdf]

ESM 2

Results for all analyses regarding order effects between T2 and T3

Table S1

Differences in the subjective control measures between Testing Session 2 and Testing Session 3 in the Footbonaut task (motivation assessed before, all other measures assessed after performing the task)

	T2		T3				
	M	*SD*	*M*	*SD*	*t*(*26*)	*p*	*d*
Motivation	89.93	11.97	89.93	12.78	0.00	> .999	.000
Mental Effort	66.52	18.46	55.78	19.17	4.18	< .001	.804
Mental Fatigue	57.67	22.33	51.93	19.20	1.63	.297	.213
Physical Fatigue	66.82	19.49	62.04	20.07	1.26	.219	.242
Boredom	19.93	29.64	13.74	19.57	1.11	.279	.213

Table S2

Estimated marginal means and results of the two-way repeated measures ANOVA (Testing Session (T2 vs. T3) x Sets in the Footbonaut (S1 vs. S2 vs. S3 vs. S4)) for the performance-related measures of the Footbonaut

Variable	T2		T3		Total (main effect S)		ANOVA				
	M	*SE*	*M*	*SE*	*M*	*SE*	Effect	*df*	F	*p*	η^2_p
Accuracy											
S1	85.83	1.03	89.16	1.20	87.50	1.02	TS	1, 25	11.68	.002	.318
S2	84.01	1.53	86.28	1.04	85.15	1.13	S	3, 75	3.02	.035	.108
S3	84.14	1.62	86.27	1.32	85.20	1.23	TS x S	3, 75	0.27	.847	.011
S4	83.92	1.44	87.24	1.46	85.58	1.25					
Total (main effect TS)	84.47	1.17	87.24	1.02							
Response Time											
S1	2.26	0.04	2.28	0.04	2.27	0.03	TS	1.00, 26,00	0.47	.501	.018
S2	2.30	0.03	2.26	0.03	2.28	0.02	S	1.64, 42.54	1.72	.195	.062
S3	2.33	0.03	2.29	0.02	2.31	0.02	TS x S	1.82, 47.28	0.96	.382	.036
S4	2.26	0.02	2.27	0.02	2.27	0.02					
Total (main effect TS)	2.29	0.02	2.27	0.02							

Note. *N* = 27; TS = Testing Session (T2 vs. T3), S = Sets in the Footbonaut (S1 vs. S2 vs. S3 vs. S4).

Table S3

Estimated marginal means and results of the two-way repeated measures ANOVA (Testing Session (T2 vs. T2) x Time of Assessment (Pre vs. Post)) for the subjective control measures of the Stroop task parameters

Variable	T2		T3		Total (main effect T)		ANOVA			
	M	*SE*	*M*	*SE*	*M*	*SE*	Effect	*F*(1, 26)	*p*	η^2_p
Motivation										
Pre	76.26	4.73	77.82	4.74	77.04	4.48	TS	1.33	.259	.049
Post	67.37	5.40	70.15	5.23	68.76	5.01	T	8.45	.007	.245
Total (main effect TS)	71.82	4.73	73.98	4.53			TS x T	0.05	.825	.002
Mental Effort										
Pre	37.26	4.95	32.26	3.86	34.76	3.82	TS	2.45	.129	.086
Post	59.04	3.77	53.04	4.74	56.43	3.77	T	41.93	< .001	.617
Total (main effect TS)	48.54	3.83	42.65	3.95			TS x T	0.179	.676	.007
Mental Fatigue										
Pre	30.85	3.87	26.33	3.87	28.60	3.40	TS	3.84	.061	.129
Post	60.37	3.87	52.30	3.87	56.33	3.40	T	65.89	< .001	.717
Total (main effect TS)	45.61	3.35	39.32	3.35			TS x T	.0876	.358	.033
Physical Fatigue										
Pre	20.78	3.66	21.59	3.66	21.19	3.22	TS	0.24	.627	.009
Post	54.63	3.66	50.89	3.66	52.76	3.22	T	114.42	< .001	.815
Total (main effect TS)	37.70	3.22	36.24	3.22			TS x T	1.53	.227	.056

Note. *N* = 27; TS = Testing Session (T2 vs. T3), T = Time of Assessment (Pre vs. Post).

Table S4

Estimated marginal means and results of the two-way repeated measures ANOVA (Testing Session (T2 vs. T2) x Time of Assessment (Pre vs. Post)) for the subjective control measures of the LSPT

Variable	T2		T3		Total (main effect T)		ANOVA			
	M	*SE*	*M*	*SE*	*M*	*SE*	Effect	*F*(1, 26)	*p*	η^2_p
Motivation										
Pre	78.74	4.53	79.07	4.24	78.91	4.29	TS	1.84	.187	.066
Post	67.89	5.65	74.74	4.91	71.32	4.77	T	13.42	.001	.340
Total (main effect TS)	73.32	4.83	76.91	4.38			TS x T	2.071	.162	.074
Mental Effort										
Pre	36.67	4.00	32.26	4.00	34.46	3.41	TS	1.77	.195	.064
Post	58.41	4.00	53.04	4.00	55.72	3.41	T	51.71	< .001	.665
Total (main effect TS)	47.54	3.58	42.65	3.58			TS x T	.059	.811	.002
Mental Fatigue										
Pre	33.04	3.95	29.85	3.95	31.44	3.43	TS	3.10	.090	.107
Post	62.93	3.95	54.93	3.95	58.93	3.43	T	67.55	< .001	.722
Total (main effect TS)	47.98	3.39	42.39	3.39			TS x T	1.12	.299	.041
Physical Fatigue										
Pre	33.52	3.78	31.67	3.78	32.59	3.37	TS	0.80	.381	.030
Post	62.41	3.78	59.15	3.78	60.78	3.37	T	41.99	< .001	.618
Total (main effect TS)	47.96	2.95	45.41	3.78			TS x T	0.15	.720	.006

Note. *N* = 27; TS = Testing Session (T2 vs. T3), T = Time of Assessment (Pre vs. Post).

Table S5

Estimated marginal means and results of the two-way repeated measures ANOVA (Testing Session (T2 vs. T3) x Assessments in the Footbonaut (Baseline vs. 1 vs. 2 vs. 3 vs. 4)) for the physiological measures of the Footbonaut

Variable	T2		T3		Total (main effect T)		ANOVA				
	M	*SE*	*M*	*SE*	*M*	*SE*	Effect	*df*	*F*	*p*	η^2_p
HR											
Baseline	98.85	4.16	88.19	2.09	93.52	2.58	TS	1.00, 26.00	37.43	< .001	.590
1	178.89	1.15	167.93	2.59	173.41	1.68	T	1.47, 38.20	870.01	< .001	.971
2	177.85	1.49	167.11	2.29	172.48	1.72	TS x T	1.58, 40.97	0.01	.971	< .001
3	176.41	1.70	165.52	2.31	170.96	1.82					
4	176.15	2.18	165.78	2.62	170.96	2.22					
Total (main effect TS)	161.63	1.74	150.90	2.13							
Bla											
Baseline	2.89	0.32	2.35	0.22	2.62	0.25	TS	1.00, 26.00	44.58	> .001	.632
1	5.93	0.50	3.63	0.37	4.78	0.40	T	2.12, 55.07	34.27	< .001	.361
2	4.95	0.47	2.76	0.32	3.85	0.36	TS x T	2.96, 76.88	14.70	< .001	.361
3	3.82	0.39	2.37	0.29	3.10	0.32					
4	3.22	0.29	2.23	0.24	2.72	0.25					
Total (main effect TS)	4.16	0.35	2.67	0.26							

Note. *N* = 27; TS = Testing Session (T2 vs. T3), T = Time of Assessment in the Footbonaut (Baseline vs. 1 vs. 2 vs. 3 vs. 4).

Table S6

Estimated marginal means and results of the two-way repeated measures ANOVA (Testing Session (T2 vs. T3) x Time of Assessment (Pre vs. Post)) for the performance-related Stroop task parameters

Variable	T2		T3		Total (main effect T)		ANOVA			
	M	*SE*	*M*	*SE*	*M*	*SE*	Effect	*F*(1, 26)	*p*	η^2_p
Trials										
Pre	143.52	3.87	152.70	3.87	148.11	3.42	TS	10.99	.003	.297
Post	149.22	3.87	159.96	3.87	154.59	3.42	T	6.871	.014	.209
Total (main effect TS)	146.37	3.52	156.33	3.52			TS x T	0.140	.712	.005
Errors										
Pre	7.78	2.60	6.56	2.60	7.17	2.28	TS	0.00	.947	> .001
Post	7.00	2.60	8.48	2.60	7.74	2.28	T	0.12	.733	.005
Total (main effect TS)	7.39	2.33	7.52	2.33			TS x T	0.71	.407	.027
Response Time										
Pre	817.50	29.05	707.26	29.05	762.38	28.01	TS	50.09	< .001	.658
Post	732.86	29.05	650.42	29.05	691.64	28.01	T	35.92	< .001	.580
Total (main effect TS)	775.18	28.21	678.18	28.21			TS x T	3.72	.065	.125

Note. *N* = 27; TS = Testing Session (T2 vs. T3), T = Time of Assessment (Pre vs. Post).

Table S7

Estimated marginal means and results of the two-way repeated measures ANOVA (Testing Session (T2 vs. T3) x Time of Assessment (Pre vs. Post)) for the performance-related LSPT parameters

Variable	T2		T3		Total (main effect T)		ANOVA			
	M	*SE*	*M*	*SE*	*M*	*SE*	Effect	*F*(1, 26)	*p*	η^2_p
Movement Time										
Pre	102.11	1.10	101.42	1.48	101.76	1.20	TS	1.31	.262	.048
Post	99.04	1.29	97.79	1.00	98.41	0.98	T	13.90	< .001	.348
Total (main effect TS)	100.57	1.05	99.60	1.13			TS x T	0.14	.708	.005
Penalty Time										
Pre	1.22	1.89	1.59	3.22	1.41	2.20	TS	0.00	.953	< .001
Post	1.56	2.62	1.52	3.00	1.54	2.32	T	0.01	.925	< .001
Total (main effect TS)	1.39	2.12	1.56	2.94			TS x T	0.02	.877	< .001
Performance Time										
Pre	103.33	2.22	102.99	3.41	103.16	2.38	TS	0.07	.796	.003
Post	100.56	2.88	99.28	3.23	99.92	2.52	T	4.01	.056	.113
Total (main effect TS)	101.94	2.35	101.14	3.15			TS x T	0.13	.723	.005

Note. *N* = 27; TS = Testing Session (T2 vs. T3), T = Time of Assessment (Pre vs. Post).
