# Supplementary material for: Increasing ecological validity in mental fatigue research—A Footbonaut study
Source: Front Psychol. 2025 May 27;16:1586944. doi: 10.3389/fpsyg.2025.1586944 (PMC12149105; doi:10.3389/fpsyg.2025.1586944)
Supplement: Supplementary file 4 [file Data_Sheet_4.pdf]

ESM 3

Additional information on the results

Table S8

Estimated marginal means and results of the two-way repeated measures ANOVA (Condition (Experimental vs. Control) x Sets in the Footbonaut (S1 vs. S2 vs. S3 vs. S4)) for the performance-related measures of the Footbonaut

Variable	Experimental Condition		Control Condition		Total (main effect S)		ANOVA				
	M	*SE*	*M*	*SE*	*M*	*SE*	Effect	*df*	*F*	*p*	η^2_p
Accuracy											
S1	88.24	1.12	86.34	1.15	87.29	1.00	C	1, 26	7.38	0.01	0.22
S2	82.85	1.28	87.03	1.20	84.94	1.11	S	3, 78	2.88	0.04	0.10
S3	82.88	1.36	87.54	1.37	85.21	1.19	C x S	3, 78	7.42	< .001	0.22
S4	84.49	1.50	86.60	1.33	85.55	1.21					
Total (main effect C)	84.62	1.09	86.88	1.05							
Response Time											
S1	2.25	0.05	2.28	0.02	2.27	0.03	C	1.00, 26.00	0.16	0.69	0.01
S2	2.30	0.03	2.27	0.03	2.28	0.02	S	1.64, 42.54	1.72	0.20	0.06
S3	2.33	0.03	2.29	0.03	2.31	0.02	C x S	1.81, 47.15	1.46	0.24	0.05
S4	2.26	0.02	2.27	0.03	2.27	0.02					
Total (main effect C)	2.29	0.02	2.28	0.02							

Note. N = 27; C = Condition (Experimental vs. Control), S = Sets in the Footbonaut (S1 vs. S2 vs. S3 vs. S4).

Figure S1

Condition-specific differences in the Accuracy of the Footbonaut tasks

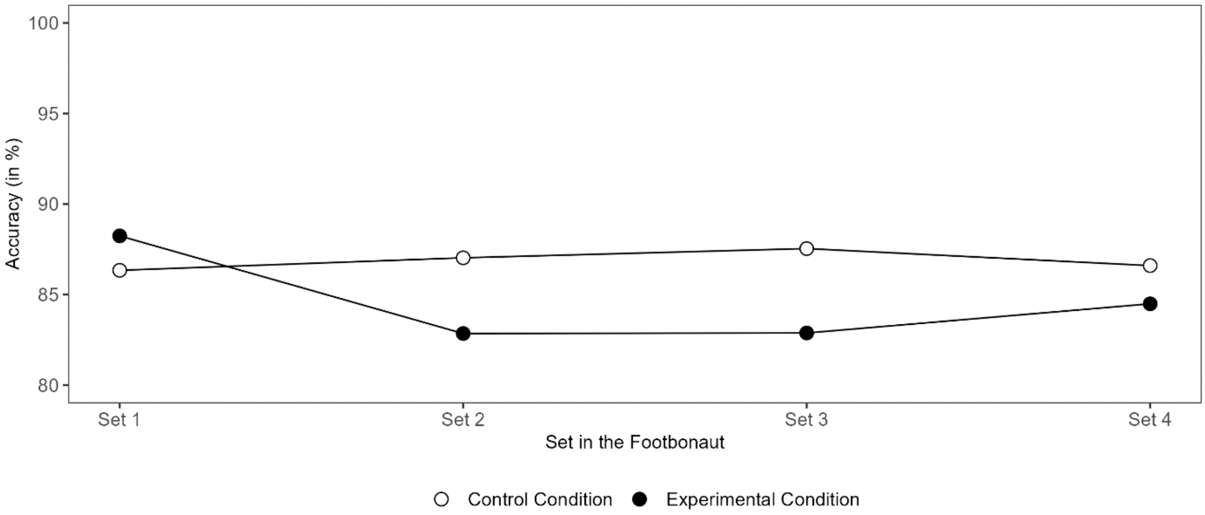

Table S9

Estimated marginal means and results of the two-way repeated measures ANOVA (Condition (Experimental vs. Control) x Time of Assessment (Pre vs. Post)) for the subjective control measures of the Stroop task parameters

Variable	Experimental Condition		Control Condition		Total (main effect T)		ANOVA			
	M	*SE*	*M*	*SE*	*M*	*SE*	Effect	*F*(1, 26)	*p*	η^2_p
Motivation										
Pre	75.37	5.03	78.70	5.03	77.04	4.75	C	< .001	.977	< .001
Post	70.37	5.03	67.15	5.03	68.76	4.75	T	8.46	.007	.245
Total (main effect C)	72.87	4.64	72.93	4.64			C x T	1.51	.230	.055
Mental Effort										
Pre	33.11	3.92	36.41	4.93	34.76	3.82	C	0.66	.425	.025
Post	54.93	4.40	57.93	4.25	56.43	3.77	T	41.93	< .001	.617
Total (main effect C)	44.02	3.81	47.17	4.03			C x T	0.01	.945	< .001
Mental Fatigue										
Pre	27.96	3.13	29.22	4.30	28.59	3.21	C	0.38	.542	.014
Post	54.85	4.17	57.82	3.98	56.33	3.57	T	65.89	< .001	.717
Total (main effect C)	41.41	3.27	43.52	3.52			C x T	0.20	.662	.007
Physical Fatigue										
Pre	20.22	3.66	22.15	3.66	21.19	3.22	C	1.06	.313	.039
Post	50.70	3.66	54.82	3.66	52.76	3.22	T	114.42	< .001	.815
Total (main effect C)	35.46	3.21	38.48	3.21			C x T	0.337	.567	.013

Note. *N* = 27; C = Condition (Experimental vs. Control), T = Time of Assessment (Pre vs. Post).

Table S10

Estimated marginal means and results of the two-way repeated measures ANOVA (Condition (Experimental vs. Control) x Time of Assessment (Pre vs. Post)) for the subjective control measures of the LSPT

Variable	Experimental Condition		Control Condition		Total (main effect T)		ANOVA			
	M	*SE*	*M*	*SE*	*M*	*SE*	Effect	*F*(1, 26)	*p*	η^2_p
Motivation										
Pre	78.00	4.47	79.81	4.30	78.91	4.29	C	0.10	.758	.004
Post	71.37	5.04	71.26	5.61	71.32	4.77	T	13.42	.001	.340
Total (main effect C)	74.69	4.51	75.54	4.73			C x T	0.17	.685	.006
Mental Effort										
Pre	35.33	3.88	32.63	3.54	33.98	3.09	C	0.07	.788	.003
Post	54.19	4.12	55.07	3.87	54.63	3.64	T	47.81	< .001	.648
Total (main effect C)	44.76	3.65	43.85	3.26			C x T	1.14	.295	.042
Mental Fatigue										
Pre	29.41	4.10	33.48	3.87	31.44	3.30	C	1.02	.321	.038
Post	57.63	4.20	60.22	3.74	58.93	3.56	T	67.55	< .001	.722
Total (main effect C)	43.52	3.76	46.85	3.05			C x T	0.102	.752	.004
Physical Fatigue										
Pre	30.63	3.76	34.56	3.76	32.59	3.37	C	2.04	.165	.073
Post	58.74	3.76	62.82	3.76	60.78	3.37	T	41.99	< .001	.618
Total (main effect C)	44.69	2.93	48.69	2.93			C x T	.002	.968	< .001

Note. *N* = 27; C = Condition (Experimental vs. Control), T = Time of Assessment (Pre vs. Post).

Table S11

Estimated marginal means and results of the two-way repeated measures ANOVA (Condition (Experimental vs. Control) x Assessments in the Footbonaut (Baseline vs. 1 vs. 2 vs. 3 vs. 4)) for the physiological measures of the Footbonaut

Variable	Experimental Condition		Control Condition		Total (main effect T)		ANOVA				
	M	*SE*	*M*	*SE*	*M*	*SE*	Effect	*df*	*F*	*p*	η^2_p
HR											
Baseline	92.04	2.48	95.00	4.19	93.52	2.58	C	1.00, 26.00	2.48	0.13	.087
1	171.52	2.64	175.30	1.76	173.41	1.68	T	1.47, 38.20	870.01	< .001	.971
2	170.11	2.57	174.85	1.63	172.48	1.72	C x T	1.58, 40.95	0.15	.809	.006
3	168.85	2.73	173.07	1.65	170.96	1.82					
4	168.52	2.90	173.41	2.19	170.96	2.22					
Total (main effect C)	154.21	2.36	158.33	1.96							
Bla											
Baseline	2.53	0.29	2.72	0.26	2.62	0.25	C	1.00, 26.00	0.67	.419	.025
1	4.55	0.50	5.01	0.48	4.78	0.40	T	2.12, 55.07	34.27	< .001	.569
2	3.73	0.45	3.98	0.46	3.85	0.36	C x T	2.17, 56.43	0.24	.806	.009
3	2.99	0.35	3.20	0.40	3.10	0.32					
4	2.53	0.23	2.92	0.33	2.72	0.25					
Total (main effect C)	3.27	0.34	3.56	0.35							

Note. *N* = 27; C = Condition (Experimental vs. Control), T = Time of Assessment in the Footbonaut (Baseline vs. 1 vs. 2 vs. 3 vs. 4).

Table S12

Estimated marginal means and results of the two-way repeated measures ANOVA (Condition (Experimental vs. Control) x Time of Assessment (Pre vs. Post)) for the performance-related Stroop task parameters

Variable	Experimental Condition		Control Condition		Total (main effect T)		ANOVA			
	M	*SE*	*M*	*SE*	*M*	*SE*	Effect	*F*(1, 26)	*p*	η^2_p
Trials										
Pre	146.93	3.99	149.30	3.99	148.11	3.42	C	0.02	.894	< .001
Post	156.26	3.99	152.93	3.99	154.59	3.42	T	6.87	.014	.209
Total (main effect C)	151.59		151.11	3.65			C x T	2.01	.168	.072
Errors										
Pre	8.85	3.64	5.48	0.85	7.17	2.04	C	0.50	.488	.019
Post	7.41	2.70	8.074	2.37	7.74	2.50	T	0.12	.733	.005
Total (main effect C)	8.13	2.98	6.78	1.40			C x T	1.64	.211	.059
Response Time										
Pre	763.63	30.57	761.13	30.57	762.38	28.01	C	0.02	.888	< .001
Post	687.07	30.57	696.21	30.57	691.64	28.01	T	35.92	< .001	.580
Total (main effect C)	725.35	29.75	728.67	29.75			C x T	0.58	.452	.022

Note. *N* = 27; C = Condition (Experimental vs. Control), T = Time of Assessment (Pre vs. Post).

Table S13

Estimated marginal means and results of the two-way repeated measures ANOVA (Condition (Experimental vs. Control) x Time of Assessment (Pre vs. Post)) for the performance-related LSPT parameters

Variable	Experimental Condition		Control Condition		Total (main effect T)		ANOVA			
	M	*SE*	*M*	*SE*	*M*	*SE*	Effect	*F*(1, 26)	*p*	η^2_p
Movement Time										
Pre	101.19	1.37	102.33	1.23	101.76	1.20	C	0.27	.609	.010
Post	98.54	1.21	98.29	1.11	98.41	0.99	T	13.90	< .001	.348
Total (main effect C)	99.87	1.19	100.31	0.99			C x T	0.94	.342	.035
Penalty Time										
Pre	> 0.01	1.82	2.59	3.25	1.30	2.20	C	> 0.01	.984	> .001
Post	2.74	2.87	0.26	2.75	1.50	2.32	T	0.02	.882	> .001
Total (main effect C)	1.37	2.23	1.43	2.87			C x T	4.36	.047	.143
Performance Time										
Pre	101.30	2.08	105.02	3.46	103.16	2.38	C	0.07	.873	.001
Post	101.28	2.84	98.56	3.25	99.92	2.52	T	4.01	.056	.133
Total (main effect C)	101.29	2.32	101.79	3.18			C x T	7.79	.010	0.230

Note. *N* = 27; C = Condition (Experimental vs. Control), T = Time of Assessment (Pre vs. Post).
